# TP53 Expression Status Alters Hemoglobinization and Ferroptosis Sensitivity in K-562 Cells

**DOI:** 10.3390/ijms26178359

**Published:** 2025-08-28

**Authors:** Cameron Cardona, Madelyne Young, McKale Montgomery

**Affiliations:** 1 Department of Nutritional Sciences, Oklahoma State University, Stillwater, OK 74078, USA; cameron.cardona@okstate.edu (C.C.); madelyne.young@okstate.edu (M.Y.); 2 Department of Nutritional Sciences, Texas Christian University, Fort Worth, TX 76109, USA

**Keywords:** lipidomics, erythropoiesis, ribosomopathies, iron metabolism

## Abstract

Activation of TP53 signaling during ribosome biogenesis is an essential part of erythroid development, whereas the pathologic activation of TP53 in ribosomopathies such as Diamond-Blackfan anemia (DBA) and del (5q) myelodysplastic syndrome (MDS) prevents the normal expansion of erythroid precursors. TP53 can also be linked to the pathogenesis of DBA and MDS via ferroptosis, a form of iron-mediated cell death propagated by excess polyunsaturated fatty acid-containing oxidizable phospholipids and loss of lipid peroxide repair capacity. The primary objective of this work was to establish how overexpression and mutation of the TP53 gene influences lipid composition, erythroid differentiation, and ferroptosis sensitivity in K-562 cells, an in vitro model for studying erythropoiesis. Employing a reverse genetics approach, we generated four isogenic cell lines that either lacked functional TP53 expression, expressed wild-type (WT) TP53, or expressed one of the two most common TP53 mutation types, R175H or R282W. We then utilized non-targeted lipidomics to quantify and identify changes in specific lipid species that occur with induction of WT and mutant TP53 expression. We also analyzed differences in gene expression, ferroptosis sensitivity, and hemoglobinization by qPCR, CCK-8 cytotoxicity assay, and o-dianisidine staining, respectively. The abundance of 337 distinct lipid species was impacted by induction of WT TP53 expression compared to K-562 cells expressing a nonfunctional P53 protein. Yet only 17 lipid compounds were differentially impacted between cells expressing WT TP53 and either of the mutant TP53 genes tested. Similarly, while the TP53 null K-562 cells displayed modest sensitivity to ferroptosis, cells expressing both WT and mutant TP53 genes were remarkably resistant to ferroptosis. However, terminal differentiation and hemoglobinization were significantly impacted in R175H mutant TP53-expressing K-562 cells. Findings from this work provide novel insights into the role of TP53 in lipid metabolism and terminal erythropoiesis.

## 1. Introduction

The highly controlled arrest of ribosome biogenesis and subsequent activation of the TP53 transcriptional program is an essential component of normal erythroid development [1,2]. Defects in this pathway can lead to P53 protein accumulation, cell cycle arrest, and ultimately an erythroid failure phenotype that is characteristic of patients with ribosomopathies such as Diamond-Blackfan anemia (DBA) and del (5q) myelodysplastic syndrome (MDS) [2]. During maturation, developing red blood cells must also undergo a metabolic shift from oxidative phosphorylation to glycolysis in addition to generating and maintaining a specific cell membrane lipid composition. Failure to do so is exemplified by the pathologic disturbances of red cell membranes in several genetic disorders [3]. While TP53 has established roles in the coordination of both cellular lipid and glucose metabolism, its capacity to influence lipid composition during terminal erythroid differentiation has yet to be fully elucidated.

The pathologic activation of TP53 in ribosomopathies such as DBA and MDS is significant because both TP53 overexpression and mutations in TP53 are associated with impaired erythropoiesis, resistance to conventional therapies, and worsened overall survival rates in these patients [1,4]. These distinct clinical outcomes have primarily been attributed to the hyperactivation of TP53 functions that negatively impact cell cycle, DNA repair, and apoptosis pathways. However, mutations in TP53 can also alter cellular iron distribution [5] and significantly influence how cells respond to changes in iron availability [6]. As proper iron balance is crucial for the maintenance of normal hematopoiesis and erythropoiesis, there is a critical need to understand how iron homeostasis and erythroid differentiation are maintained in cells expressing mutant TP53.

TP53 can also be linked to the pathogenesis of DBA and MDS via ferroptosis, a form of iron-mediated cell death [7,8,9]. In addition to the availability of redox-active iron, ferroptosis is also dependent upon the oxidation of polyunsaturated fatty acid-containing phospholipids and/or the loss of lipid peroxide repair capacity by glutathione peroxidase 4 (GPX4) [10]. Our lab has recently shown that induction of distinct TP53 mutation types can lead to impaired fatty acid desaturation capacity and reduced GPX4 activity [11] and are associated with increased ferroptosis sensitivity [12]. These findings are significant because GPX4 loss in erythroid cells can impair reticulocyte maturation and disrupt iron homeostasis during erythropoiesis in both ferroptosis-dependent and -independent manners [13,14,15]. In DBA and MDS patients, excess heme can block erythropoiesis, and the repeated red blood cell transfusions to treat the subsequent anemia commonly leads to iron overload. Thus, understanding how TP53 expression status influences erythroid cell sensitivity to ferroptosis is significant because it can inform our understanding of how patients will respond to conventional therapies.

The primary objective of this work was to establish how TP53 expression and mutation status influence erythroid cell lipid composition, ferroptosis sensitivity, and differentiation in an in vitro model of erythropoiesis. The central hypothesis was that mutant TP53-dependent disruptions in antioxidant and lipid handling pathways limit the iron handling capacity and maturation of erythroid precursors. Our results indicate that, irrespective of TP53 mutation type, induction of TP53 expression has a profound impact on erythroid lipid composition, heme metabolism-related mRNA expression, and ferroptosis resistance in K-562 cells.

## 2. Results

### 2.1. Induction of TP53 Expression Dramatically Alters K-562 Cell Lipid Composition

To obtain a comprehensive and representative overview of the TP53 lipidome, we utilized non-targeted lipidomics to measure the impact of inducing the expression of TP53 on cellular lipid composition. The K-562 cell line was selected because they are a validated in vitro model of erythropoiesis [16], and because they harbor an inactivation mutation in the TP53 gene that leads to the expression of a truncated, nonfunctional version of the P53 protein [17]. We then transfected the cells with either an empty vector plasmid (Null), or a plasmid containing either wild-type TP53 or one of two of the most common TP53 mutation types. These mutation types were selected because they occur at a much higher frequency than other TP53 mutation types [18]. They are also observed at a rate as high as 20% MDS patients [19] and are associated with poor clinical outcomes in these patients [4]. After adjusting for the False Discovery Rate, and normalizing to total lipids, 568 distinct lipid compounds were identified in all four cell lines (Supplementary Table S2).

**Figure 1:**
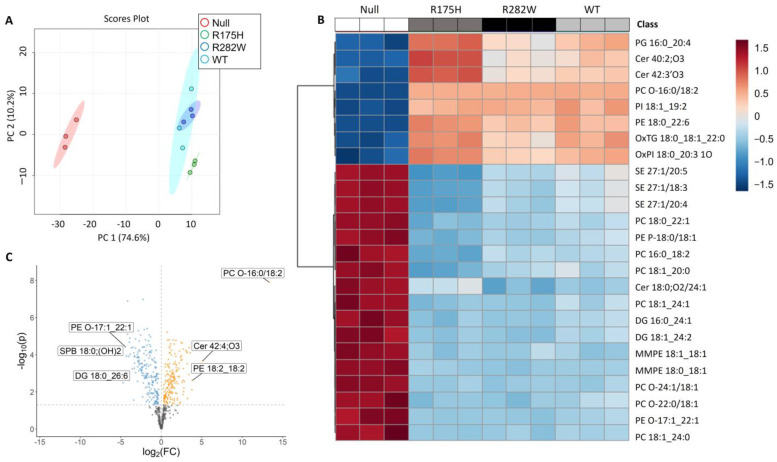
Comprehensive analysis of the TP53-dependent changes in K-562 cell lipid composition. (**A**) Principal component analysis indicating the largest differences between K-562 cells expressing a nonfunctional P53 protein (Null) and K-562 cells overexpressing either a WT, R175H, or R282W P53 protein. (**B**) Heat map indicating the relative intensities of the top 25 most changed lipid species as indicated by one-way ANOVA. (**C**) Volcano plots summarizing significantly (*p* < 0.05) downregulated (blue) and upregulated (yellow) lipid species between K-562 cells null for P53 and K-562 cells expressing WT P53.

Principal component analysis revealed that induction of TP53 expression alone, regardless of mutation status, was sufficient to generate marked separation of lipid composition from the TP53 null cells (Figure 1A). Lipid profiles between WT- and mutant TP53-expressing cells were highly similar. The distinction in lipid profiles between the TP53 null cells from their WT- and mutant TP53-expressing counterparts is further illustrated in the heat map (Figure 1B) which highlights the 25 most differentially expressed lipid species. Lastly, the similarities in lipid profiles between WT- and mutant TP53-expressing cell types were confirmed via one-way-ANOVA which revealed only a modest profile shift (17 differentially expressed lipid species) between the WT- and R175H-expressing cells and no significant differences in lipid species between the WT- and R282W TP53-expressing cells (Supplementary Table S3). These findings are somewhat surprising because, at least in other cell types, induction of R175H and R282W mutations have been shown to cause dramatic changes in the cell transcriptome and proteome [11,18], whereas in K-562 cells, induction of TP53 mutations did not significantly alter the cell lipidome compared to induction of WT TP53 expression, suggesting that the functionality of TP53-mediated regulation of cell lipid metabolism is highly conserved.

### 2.2. Lipids Involved in Choline Metabolism, Sphingolipid Metabolism, Glycerophospholipid Metabolism, and Ferroptosis, Among Others Differentially Expressed Following Induction of TP53

We then shifted our focus to understanding the biologic significance of the changes in K-562 cell lipid composition upon expression of TP53, concentrating particularly on the effects of WT TP53. A total of 337 compounds were significantly altered upon induction of WT TP53 expression compared to K-562 null cells (Figure 1C). The differentially expressed lipid species are involved in a variety of diverse signaling pathways ranging from glycerophospholipid metabolism to sphingolipid metabolism (Table 1). Though not statistically significant (*p* = 0.06), given the established roles for TP53 and lipid species composition in ferroptosis, we chose to further investigate the influence of TP53 expression status on K-562 ferroptosis susceptibility.

### 2.3. Induction of WT and Mutant TP53 Expression Increases Ferroptosis Resistance but Not GPX4 mRNA Expression in K-562 Cells

**Figure 2:**
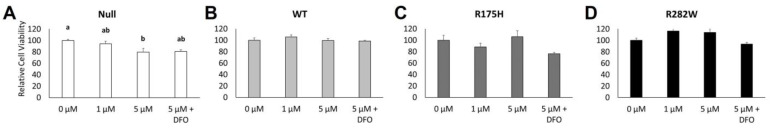
WT and mutant TP53 expression increase ferroptosis resistance in K-562 cells. Relative cell viability of K-562 cells expressing (**A**) nonfunctional P53 protein (Null), or K-562 cells transfected with (**B**) WT TP53, (**C**) R175H TP53, or (**D**) R282W TP53 in response to treatment with the indicated doses of RSL3 for 48 h. To demonstrate that the Null cells were dying from ferroptosis, cells were rescued by treatment with the iron chelator, DFO. Differing superscripts indicate statistically significant differences in cell viability as analyzed by one-way ANOVA using Tukey’s post hoc test (*p* < 0.05).

Previous work has established that K-562 cells are resistant to ferroptosis induction via the system x_c−_ inhibitor, erastin [20]. Thus, we tested differences in K-562 cell ferroptosis sensitivity by treating cells with RSL3, which induces ferroptosis by directly inhibiting GPX4, effectively destroying the cell’s antioxidant defense system [21]. K-562 cells expressing the nonfunction TP53 mutant (Null) displayed only modest sensitivity to treatment with RSL3 (Figure 2A). The small decrease in cell viability was partially rescued by treatment with the iron chelator deferoxamine (DFO), which indicates ferroptosis was indeed the cause of cell death. Remarkably, induction of WT TP53 as well as R175H and R282W mutant TP53 conferred complete insensitivity to RSL3 treatment at each of the doses tested (Figure 2B–D).

**Figure 3:**
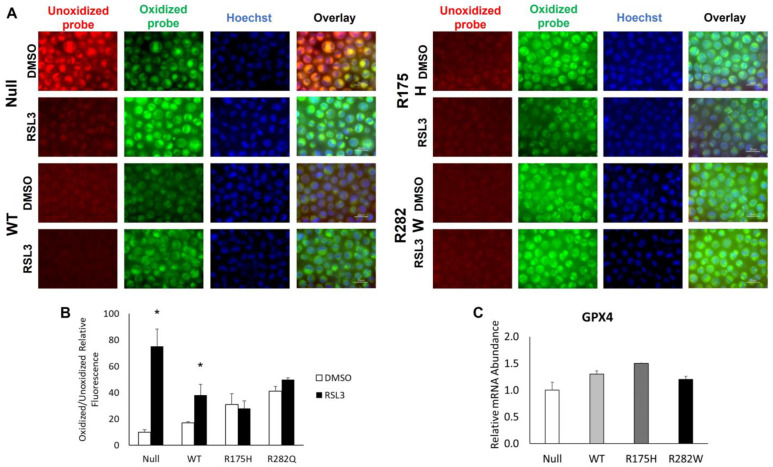
WT- and mutant TP53-expressing K-562 cells are less sensitive to RSL3-mediated lipid peroxidation. (**A**) Unoxidized (red) and oxidized (green) lipid species were visualized by staining with BODIPY 581/591 C11. (**B**) Changes in relative amounts of oxidized probe were quantified using ImageJ software, version 1.54g, following normalization to Hoechst nuclear staining (blue). (**C**) Relative expression of GPX4 mRNA in each of the distinct TP53-expressing K-562 subtypes. * Denotes significance from DMSO treated control, *p* < 0.05.

To fully demonstrate that the dose of RSL3 used was indeed sufficient to induce ferroptosis, we measured differences in lipid peroxidation before and after treatment with 5 µM RSL3. Consistent with our viability findings, the 5 µM RSL3 dose was sufficient to significantly increase lipid peroxidation in TP53 null K-562 cells (Figure 3B). Lipid peroxidation was also significantly increased in the WT TP53-expressing cells, albeit to a lesser extent, whereas no measurable changes in lipid peroxidation were observed in either the R175H- or R282W TP53-expressing mutants (Figure 3B). To investigate a potential mechanism contributing to such strong ferroptosis resistance in TP53-expressing cell lines, we measured basal mRNA abundance of the potent antioxidant, GPX4, but found it was not significantly altered by induction of TP53 expression (Figure 3C). Collectively, such findings indicate that TP53-mediated alterations in lipid metabolism do not render erythroid cells more susceptible to ferroptotic cell death, but rather expression of both WT and mutant TP53 may actually confer ferroptosis resistance.

### 2.4. Hemoglobinization and Heme Metabolism-Related mRNA Expression Are Differentially Altered by Induction of WT and Mutant TP53 Expression

**Figure 4:**
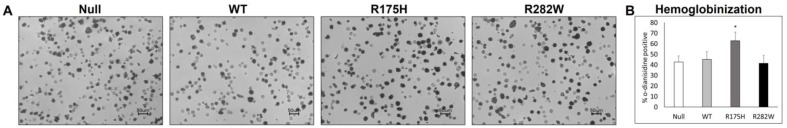
K-562 cells expressing the R175H TP53 mutation exhibit higher rates of hemoglobinization. Representative images of o-dianisidine stained K-562 cells expressing (**A**) nonfunctional P53 protein (Null), or K-562 cells expressing either WT, R175H, or R282W P53. Black cells are positively stained for hemoglobin. (**B**) Quantification of positively stained K-562 cells relative to the total number of cells from three separate experiments with at least 3 biologic replicates/cell type. * Denotes statistical significance, *p* < 0.05.

Given the role of TP53 and lipid metabolism in terminal erythropoiesis [1] we next sought to determine how induction of WT and mutant TP53 would influence hemoglobinization and mRNA expression patterns following differentiation. Following 3 days of incubation with 30 µM hemin, K-562 cells expressing the R175H mutant exhibited the highest level hemoglobinization as indicated by positive o-dianisidine staining (Figure 4A,B). Hemoglobinization was not different between the K-562 cells expressing the nonfunctional P53 protein (Null), WT TP53 or R282W TP53.

**Figure 5:**
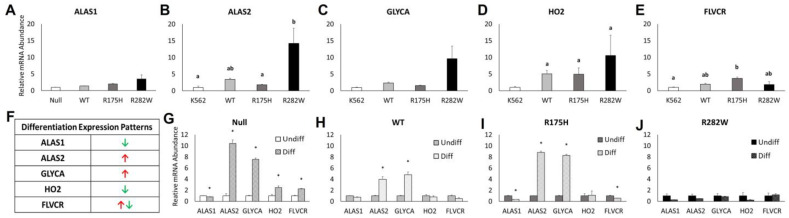
TP53 status significantly impacts the expression of genes involved in heme metabolism and hemin-induced differentiation. Relative mRNA expression of (**A**) 5-aminolevulinate synthase 1 (ALAS1), (**B**) ALAS2, (**C**) glycophorin A (GLYCA), (**D**) heme oxygenase 2 (HO2), and (**E**) feline leukemia virus subgroup C receptor (FLVCR) in K-562 cells expressing a nonfunctional P53 protein (Null), or K-562 cells transfected with WT P53, R175H, or an R282W P53. (**F**) Anticipated expression pattern changes (green down arrow = decreased; red up arrow = increased) in heme-metabolizing genes following differentiation. (**G**–**J**) Relative mRNA expression of ALAS1, ALAS2, GLYCA, HO2, and FLVCR following 72 h of hemin-induced differentiation (diff) compared to their undifferentiated (undiff) counterparts in the indicated TP53 expression subtypes. Differing superscripts indicate statistically significant differences in cell viability as analyzed by one-way ANOVA using Tukey’s post hoc test (*p* < 0.05). * Denotes statistical significance between differentiated and undifferentiated cells within a given TP53 expression type.

To determine if differences in hemoglobinization were due to differences in heme metabolism we measured the mRNA expression of the heme biosynthetic genes, 5-aminolevulinate synthase 1 (ALAS1) and its isoform ALAS2, the red cell membrane associated protein, glycophorin A (GLYCA), heme oxygenase 2 (HO2), and the heme exporter, feline leukemia virus subgroup C receptor (FLVCR). Induction of both WT and mutant TP53 expression was enough to significantly increase the expression of heme-metabolizing genes, though induction of the R282W TP53 mutation type had the most pronounced effect (Figure 5A–E).

Importantly, these mRNA have previously been shown to have predictable expression patterns during K-562 cell terminal differentiation ([22] and Figure 5F). Thus, we sought to determine how mRNA expression patterns were influenced by both TP53 expression and mutation status following K-562 cell differentiation. In general, the TP53 null K-562 cells, as well as the WT- and R175H TP53-expressing cells exhibited anticipated changes in heme-metabolizing mRNA expression patterns (Figure 5G–I). However, the magnitude of those changes varied amongst the three cell types. Moreover, the R282W TP53-expressing cells, which had higher basal expression levels in each gene tested, did not exhibit the anticipated changes in heme-metabolizing genes following differentiation (Figure 5J). Altogether, these data indicate that both TP53 expression and TP53 mutation status distinctly alter the K-562 cell response to hemin-induced differentiation.

## 3. Discussion

The coordination of ribosome biogenesis and arrest with TP53 activation is critical for erythroid differentiation [1], whereas disruptions in assembly, coupled with pathologic activation of TP53, prevent normal erythroid expansion and may increase susceptibility to ferroptosis [1,23]. The generation and maintenance of specific lipid compositions in cell membranes is also essential for terminal erythropoiesis [24] and is dependent upon functional TP53 signaling pathways [25]. Thus, we investigated the influence of TP53 mutation status on erythroid cell lipid composition, differentiation, and ferroptosis sensitivity using K-562 cells as an in vitro model of erythropoiesis.

While a variety of pathways were identified through our lipid enrichment pathway analysis as being significantly impacted, 30 of the 160 lipid species that were upregulated following the induction of WT TP53 expression are classified as phosphatidylethanolamines (PEs). PEs are the second most abundant phospholipid in mammalian cells and are associated with numerous pleiotropic functions ranging from the coordination of cell membrane topology to mitochondrial function [26]. Notably, altered fatty acid incorporation into PE has been shown to contribute to hemolysis in spur cell anemia [27] and PE oxidation is a critical initiating event in ferroptosis signaling [28]. That, coupled with the established connections between TP53 and ferroptosis, provoked us to further investigate how TP53 expression status influenced ferroptosis in K-562 cells. However, the increase in PE species abundance observed in TP53 expressing K-562 cells was not associated with increased ferroptosis sensitivity, albeit K-562 cells themselves are not particularly sensitive to ferroptosis.

For example, wild-type K-562 cells, which express a nonfunctional TP53 protein are resistant to system x_c_^−^ inhibition (i.e., erastin treatment) [20] and are only moderately sensitive to GPX4 inhibition (i.e., RSL3 treatment; Figure 2A). Unexpectedly, overexpression of both WT and mutant TP53 appeared to confer ferroptosis resistance. This effect was not due to an increase in GPX4 mRNA transcription (Figure 3C), and we have previously shown that changes in GPX4 activity do not fully explain TP53-mediated changes in ferroptosis sensitivity [11]. It is important to note that TP53 has been shown to both promote [29] and suppress ferroptosis [8], and its role as a ferroptosis gatekeeper is largely context-dependent and varies in different biological settings [30].

Generally, in cancer, TP53-mediated ferroptosis induction is an essential tumor suppressing mechanism [29]. However, loss of TP53 has also been shown to increase ferroptosis sensitivity in colorectal cancer cell lines [31] and chronic TP53 activation has been shown to delay its onset [8]. In the present study, we did not examine cell viability beyond 48 h, so it may be that induction of TP53 expression merely delayed the onset of ferroptosis rather than conferring complete resistance. Collectively though, our findings further highlight that TP53-mediated ferroptosis in a given cell type and biologic context needs to be individually and experimentally determined.

In erythropoiesis, the TP53-dependent role in terminal differentiation is much less nebulous. During normal erythroid development, ribosome biogenesis arrest leads to the activation of a TP53-dependent transcriptional program that drives erythroid gene expression [1]. In ribosomopathies such as Diamond-Blackfan anemia and MDS, disruptions in ribosome assembly promote the pathologic activation of TP53 and the prevention of normal erythroid expansion. Herein, we hypothesized that overexpression of mutant TP53, which is often pathologically activated [32], would interfere with K-562 cell hemoglobinization and differentiation.

Following three days of hemin-mediated differentiation, only ~50% of K-562 cell expressing the nonfunctional TP53, as well as WT- and R282W TP53 mutant-expressing cells had undergone erythroid induction (Figure 4), whereas ~70% of the K-562 expressing the R175H cells stained positive for hemoglobin. This response did not appear to be due to differences in basal heme metabolism as the expression of HO2, FLVCR, ALAS1, and ALAS2 were not different between WT- and R175H TP53-expressing subtypes. Rather, the R282W subtypes had the highest basal expression of HO2 and ALAS2 as well as the red cell membrane component GLYCA. Thus, we next asked whether TP53 expression status instead influenced the cellular response to heme during hemin-induced differentiation.

During normal K-562 cell erythropoiesis, high levels of intracellular heme are expected to lead to reduced expression of HO2 as well as the heme biosynthetic enzyme ALAS1, while expression of the ALAS2 isoform significantly increases [22]. This corresponds with an increase in the expression of GLYCA and a transient rise and fall in the expression of the heme exporter, FLVCR [22]. Upon differentiation, the Null and WT- and R175H TP53- expressing K-562 subtypes displayed similar changes in predicted mRNA expression patterns, but the R175H phenotype fell most in-line with the predicted pattern and had the highest degree of hemoglobinization (Figure 5). However, the R282W subtype displayed none of the predicted mRNA differentiation patterns and still had similar levels of hemoglobinization as the Null and WT TP53-expressing cells. Thus, the degree of changes in heme-metabolism-related mRNA expression patterns did not correspond with our observed changes in hemoglobinization. Future studies should examine the impact of TP53 expression status on TP53-specific transcriptional targets related to erythroid differentiation.

Both a major strength and limitation of this work was the use of a single isogenic cell line. By eliminating the variability associated with different genetic backgrounds in multiple cell lines, the findings presented herein can be attributed solely to differences in TP53 expression status. In doing so, we have identified novel roles for TP53 in the maintenance of erythroid cell lipid composition and hemoglobinization. However, given the context-dependent functions of TP53 in cellular metabolism and ferroptosis execution, the translatability of these findings to other erythroid cell types should be the subject of future investigations. Moreover, as distinct TP53 mutation types can also display distinct ferroptotic phenotypes [12,31,33], the TP53 mutation type needs to be considered as well.

## 4. Materials and Methods

### 4.1. Cell Culture and Creation of Stable WT and Mutant TP53-Expressing Cell Lines

K-562 cells (CCL-243) expressing a truncated, nonfunctional p53 protein [17] were obtained from the American Type Cell Culture Collection (ATCC; Manassas, VA, USA) and cultured according to the manufacturer’s instructions in 10-1040-CV RPMI with L-Glutamine (Corning; Corning, NY, USA) supplemented with 10% FBS (Atlanta Biologicals; Norcross, GA, USA), 100 IU/mL penicillin, and 100 ug/mL streptomycin (Corning). Isogenic cell lines were created by transfecting an empty pcDNA5/TO plasmid (Null) or a pcDNA5/TO plasmid containing either the wild-type (WT) TP53 gene or a copy of the TP53 gene containing one of the most common TP53 hotspot mutations (R175H or R282W). Transfections were performed using the TransIT-X2 Dynamic Delivery System (Mirus Bio; Madison, WI, USA) and Opti-MEM reduced serum media (ThermoFisher, Waltham, MA, USA). Selection of positively transfected cells was accomplished by passaging cells at low confluency into media containing 100 µg/mL hygromycin. Stably transfected cells were maintained by culturing in hygromycin-containing media for the duration of the studies.

### 4.2. Lipid Extraction

Lipid extraction, mass spectrometry, and analysis of data for the lipidomics experiments were performed at the University of Utah Lipidomics Core facility. Isogenic cell lines were grown to confluency in 100 cm flasks, pelleted in microcentrifuge tubes, and frozen at −80 °C before extracting lipids according to Maytash et al. [34]. Pellets were resuspended in LC-MS grade MeOH (Burdick and Jackson, Muskegon, MI, USA) containing Avanti SPLASH LIPIDOMIX (d4-succinate, d-9 carnitine) internal standards and LC-MS grade methyl tert-butyl ether (Fisher Scientific, Hampton, NH, USA). Once resuspended, samples were sonicated for 1 min, and incubated in ice for 1 h, mixing briefly by vortexing every 15 min prior to induction of phase separation through the addition of PBS. Samples were then vortexed for 20 s, incubated for 10 min at room temperature, and spun at 14,000× *g* for 10 min at 4 °C. The upper and lower phases were separately selected, and the lower aqueous phase was re-extracted with MTBE:MeOH:H_2_O (10:3:2). The upper organic phase was evaporated to dryness under a vacuum. Samples were then resuspended in mobile phase B solution and transferred to an LC/MS insert (Agilent, Santa Clara, CA, USA).

### 4.3. Mass Spectrometry Analysis

Lipid samples went through a 2.1 × 100 mm; 1.7 µM UPLC CSH C18 column attached to a 5 × 2.1 mm; 1.7 µM UPLC CSH C18 VanGuard precolumn (Acquity. Milford, MA, USA) at 65 °C with an HiP 1920 Sampler (Agilent, Santa Clara, CA, USA), 1920 Infinity pump and 6545 Accurate Mass Q-TOF dual AJS-ESI mass spectrometer (Agilent, Santa Clara, CA, USA). Samples were then analyzed following randomization using both positive and negative ionization models using separate elements. For positive mode, the source gas temperature was set to 225 °C, with a drying gas flow of 11 L/min, nebulizer pressure of 40 psig, sheath gas temp of 350 °C, and sheath gas flow of 11 L/min. VCap voltage is set at 3500 V, nozzle voltage 500 V, fragmentor at 110 V, skimmer at 85 V, and octopole RF peak at 750 V. For negative mode, the source gas temperature was set to 300 °C, with a drying gas flow of 11 L/min, a nebulizer pressure of 30 psig, sheath gas temp of 350 °C, and sheath gas flow 11 L/min. VCap voltage was set at 3500 V, nozzle voltage 75 V, fragmentor at 175 V, skimmer at 75 V, and octopole RF peak at 750 V. Mobile phase A consisted of ACN:H_2_O (60:40, *v*/*v*) in 10 mM ammonium formate and 0.1% formic acid, and mobile phase B consisted of IPA:ACN:H_2_O (90:9:1, *v*/*v*/*v*) in 10 mM ammonium formate and 0.1% formic acid. For negative mode analysis, the modifiers were changed to 10 mM ammonium acetate. The chromatography gradient for both positive and negative modes started at 15% mobile phase B then increased to 30% B over 2.4 min, it then increased to 48% B from 2.4 to 3.0 min, then increased to 82% B from 3 to 13.2 min, then increased to 99% B from 13.2 to 13.8 min where it is held until 16.7 min and then returned to the initial conditions and equilibrated for 5 min. Flow was 0.4 mL/min throughout, with injection volumes of 2 µL for positive and 8 µL negative mode. Tandem mass spectrometry was conducted using iterative exclusion, the same LC gradient at collision energies of 20 V and 27.5 V in positive and negative modes, respectively.

### 4.4. Analysis of Lipidomics Data

An Agilent Mass Hunter (MH) workstation with the MH Qualitative and MH Quantitative software packages (version 12.1) were used to process data. Pooled QC and process blanks were also injected during the sample queue to ensure reliability. The LipidMatch library [35] with accurate mass and MS/MS spectral matching was used to annotate lipids. Negative and positive ionization model data was merged by lipid class and data was evaluated in Microsoft Excel where targets were parsed. Parsing criteria resulted in only lipid species with relative standard deviations less than 30% and background counts less than 30% of QC being used in analysis. The cleaned data was then normalized to class-specific internal standards before analyzing statistically.

### 4.5. Lipid Pathway Enrichment Analysis

Lipid pathway enrichment analysis was performed using the LIPEA (https://hyperlipea.org/home, accessed on 21 February 2025) web platform. Then, KEGG pathway compound names were automatically converted to Lipid Maps IDs using the R package, lipidmapsR version 1.0.4 [36], which contains data from LIPID MAPS^®^ (https://www.lipidmaps.org/, accessed on 24 February 2025) [37]. Renaming was manually verified for at least half of the compounds using LIPID MAPS, its classification system [38], and online tools [39]. These lipid maps IDs were then manually paired back to detected lipids following conversion to abbreviations using Goslin 2.0 [40].

### 4.6. Cell Proliferation and Viability Assays

Viability was inferred based on tetrazolium salt metabolism using Cell Counting Kit-8 (CCK8; Selleck Chemicals, Houston, TX, USA) according to the manufacturer’s instructions. Differences in ferroptosis sensitivity were determined by plating cells in the heme iron supplement, Hemin, for 24 h before inducing ferroptosis by inhibiting lipid peroxide repair with the potent GPX4 inhibitor [21], RSL3 (Sellek Chemicals, Houston, TX, USA). Additionally, rescue experiments were conducted using the ferroptosis inhibitor liproxstatin-1 (Lip-1) or deferoxamine (DFO), an iron chelator that can be ferroptosis protective [21].

### 4.7. Visualization and Quantification of Lipid Peroxidation

To determine differences in lipid peroxidation, cells were pelleted gently (700× *g*) at room temperature for 5 min and washed once with Hank’s Balanced Salt Solution (HBSS; Sigma-Aldrich, St. Louis, MO, USA). Following the wash, cells were incubated in a 5 µM BODIPY 581/591 C11 solution in HBSS with Hoechst stain (ThermoFisher, Waltham, MA, USA) at 1:1000 for 15 min at 37 °C. The suspension was then pelleted gently, washed again with HBSS, and plated into a culture-insert two-well glass bottom dish (Ibidi, Fitchburg, WI, USA). Images were obtained using a Keyence BZ-X700 Fluorescent Microscope (Keyence, Osaka, Japan) with a 100X objective lens under oil. Low photobleach settings and exposure times were held constant for acquisition of all images. ImageJ software version 1.54g was used to quantify differences in the ratio of red (unoxidized) and green (oxidized) lipid fluorescence intensities, which were then normalized to cell number by counting the number of Hoechst-stained nuclei.

### 4.8. Differentiation and Assessment of Hemoglobinization

K-562 cells were differentiated into an erythroid phenotype by culturing in 30 µM hemin for 72 h as indicated in [16]. Cells were then pelleted by gentle centrifugation for 10 min at 1000× *g*. Hemoglobinization was assessed by resuspending in dianisidine staining solution (0.2% dianisidine, 0.3% hydrogen peroxide, and 0.3% acetic acid in 1X PBS) followed by a light-protected 30 min incubation at room temperature. Samples were collected again by centrifugation at 500× *g* and washed twice with 1X PBS prior to imaging using a Keyence BZ-X700 microscope at 20X. Images were opened in ImageJ and both stained, and total cell counts were determined using the cell counter feature.

### 4.9. mRNA Expression Analysis

Cells were grown to confluency under standard conditions or in hemin-supplemented media to induce differentiation, as described above. Once confluent or differentiated, total RNA was precipitated using TRIzol reagent, according to manufacturer’s instructions. NanoDrop One (ThermoFisher, Waltham, MA, USA) and gel electrophoresis were used to quantify RNA concentration and verify integrity, prior to treatment with a recombinant DNase I kit (Roche). RNA was then transcribed to cDNA using SuperScript II Reverse Transcriptase (ThermoFisher, Waltham, MA, USA). Relative mRNA expression levels were detected using SYBR green chemistry on a CFX Opus 384 Real Time PCR System (Bio-Rad, Hercules, CA, USA). Relative mRNA abundance was determined by normalizing target mRNA expression to the housekeeping gene, Peptidylprolase Isomerase B (PPIB) using the 2^−∆∆Ct^ method [41]. Primer sequences can be found in Supplementary Table S1.

### 4.10. Statistics

Statistical analysis was completed using R and multiple publicly available packages [42,43,44,45,46,47,48,49]. Differences between the means of two groups were analyzed using Student’s *t*-test and differences between the means of three or more groups were analyzed using one-way ANOVA. Differences between individual group means were further identified using Tukey’s Honest Significant Difference post hoc test, using base R and the Multcompview package version 0.1-10.

## 5. Conclusions

Mutations in TP53 have previously been linked to genomic instability and progression of MDS to acute myeloid leukemia [4], but their impact on hemoglobinization and potential to contribute to the severity of macrocytic anemia in DBA and MDS patients remained unknown. In this study, we have established that induction of both WT and mutant TP53 expression influences erythroid cell lipid composition, differentiation, and ferroptosis sensitivity, each of which can play crucial roles in DBA and MDS patient outcomes [9,24]. Of particular importance, is our finding that mutant TP53 expression can influence both the expression of heme metabolism genes and rates of hemoglobinization. As heme toxicity is a major contributor to the anemia of MDS patients [9], these findings are clinically relevant because they identify unique pathways that could be therapeutically targeted to improve individual patient outcomes.

## Figures and Tables

**Table 1:** Lipid pathway enrichment analysis of the differentially expressed lipid species between P53 null and WT TP53-expressing K-562 cells.

Pathway Name	Pathway Lipids	*p*-Value	Benjamini Correction
Glycerophospholipid metabolism	26	0.000019862	0.000536263
Ether lipid metabolism	16	0.002329627	0.023079117
Sphingolipid signaling pathway	9	0.003733457	0.023079117
Glycosylphosphatidylinositol (GPI)-anchor biosynthesis	3	0.004273911	0.023079117
Autophagy–animal	4	0.008345775	0.028166992
Necroptosis	4	0.008345775	0.028166992
Choline metabolism in cancer	5	0.013581394	0.040744182
Retrograde endocannabinoid signaling	8	0.035407737	0.087988827
Sphingolipid metabolism	21	0.043529482	0.090407385
Ferroptosis	11	0.064784195	0.124940948

## Data Availability

All data generated in these studies are available in the current manuscript and its correspondingly published Supplementary Materials.

## Supplementary Materials

The following supporting information can be downloaded at https://www.mdpi.com/article/10.3390/ijms26178359/s1.

## Abbreviations

The following abbreviations are used in this manuscript:

DBA	Diamond-blackfan anemia
MDS	del(5q) myelodysplastic syndrome
GPX4	Glutathione peroxidase 4
WT	Wild-type
qPCR	Quantitative polymerase chain reaction
CCK-8	Cell counting kit 8
ALAS1	5-aminolevulinate synthase 1
ALAS2	5-aminolevulinate synthase 2
GLYCA	Glycophorin A
HO2	Heme oxygenase 2
FLVCR	Feline leukemia virus subgroup C receptor

## References

[1] Le Goff S., Boussaid I., Floquet C., Raimbault A., Hatin I., Andrieu-Soler C., Salma M., Leduc M., Gautier E.F., Guyot B. (2021). P53 Activation during Ribosome Biogenesis Regulates Normal Erythroid Differentiation. Blood.

[2] Pant V., Quintas-Cardama A., Lozano G. (2012). The P53 Pathway in Hematopoiesis: Lessons from Mouse Models, Implications for Humans. Blood.

[3] An X., Mohandas N. (2008). Disorders of Red Cell Membrane. Br. J. Haematol..

[4] Zhang L., McGraw K.L., Sallman D.A., List A.F. (2017). The Role of P53 in Myelodysplastic Syndromes and Acute Myeloid Leukemia: Molecular Aspects and Clinical Implications. Leuk. Lymphoma.

[5] Zhang F., Wang W., Tsuji Y., Torti S.V., Torti F.M. (2008). Post-Transcriptional Modulation of Iron Homeostasis during P53-Dependent Growth Arrest. J. Biol. Chem..

[6] Clarke S.L., Thompson L.R., Dandekar E., Srinivasan A., Montgomery M.R. (2019). Distinct TP53 Mutation Subtypes Differentially Influence Cellular Iron Metabolism. Nutrients.

[7] Lv Q., Niu H., Yue L., Liu J., Yang L., Liu C., Jiang H., Dong S., Shao Z., Xing L. (2020). Abnormal Ferroptosis in Myelodysplastic Syndrome. Front. Oncol..

[8] Tarangelo A., Magtanong L., Bieging-Rolett K.T., Li Y., Ye J., Attardi L.D., Dixon S.J. (2018). P53 Suppresses Metabolic Stress-Induced Ferroptosis in Cancer Cells. Cell Rep..

[9] Yang Z., Keel S.B., Shimamura A., Liu L., Gerds A.T., Li H.Y., Wood B.L., Scott B.L., Abkowitz J.L. (2016). Delayed Globin Synthesis Leads to Excess Heme and the Macrocytic Anemia of Diamond Blackfan Anemia and Del(5q) Myelodysplastic Syndrome. Sci. Transl. Med..

[10] Dixon S.J., Stockwell B.R. (2019). The Hallmarks of Ferroptosis. Annu. Rev. Cancer Biol..

[11] Cardona C.J., Hermann E.R., Kouplen K.N., Hartson S.D., Montgomery M.R. (2022). Differences in Antioxidant and Lipid Handling Protein Expression Influence How Cells Expressing Distinct Mutant TP53 Subtypes Maintain Iron Homeostasis. Cells.

[12] Thompson L.R., Oliveira T.G., Hermann E.R., Chowanadisai W., Clarke S.L., Montgomery M.R. (2020). Distinct TP53 Mutation Types Exhibit Increased Sensitivity to Ferroptosis Independently of Changes in Iron Regulatory Protein Activity. Int. J. Mol. Sci..

[13] Altamura S., Vegi N.M., Hoppe P.S., Schroeder T., Aichler M., Walch A., Okreglicka K., Hultner L., Schneider M., Ladinig C. (2020). Glutathione Peroxidase 4 and Vitamin E Control Reticulocyte Maturation, Stress Erythropoiesis and Iron Homeostasis. Haematologica.

[14] Ouled-Haddou H., Messaoudi K., Demont Y., Lopes Dos Santos R., Carola C., Caulier A., Vong P., Jankovsky N., Lebon D., Willaume A. (2020). A New Role of Glutathione Peroxidase 4 during Human Erythroblast Enucleation. Blood Adv..

[15] Rademacher M., Kuhn H., Borchert A. (2021). Expression Silencing of Glutathione Peroxidase 4 in Mouse Erythroleukemia Cells Delays In Vitro Erythropoiesis. Int. J. Mol. Sci..

[16] Zhang D., Cho E., Wong J. (2007). A Critical Role for the Co-Repressor N-CoR in Erythroid Differentiation and Heme Synthesis. Cell Res..

[17] Law J.C., Ritke M.K., Yalowich J.C., Leder G.H., Ferrell R.E. (1993). Mutational Inactivation of the P53 Gene in the Human Erythroid Leukemic K562 Cell Line. Leuk. Res..

[18] Freed-Pastor W.A., Prives C. (2012). Mutant P53: One Name, Many Proteins. Genes. Dev..

[19] Stengel A., Kern W., Haferlach T., Meggendorfer M., Fasan A., Haferlach C. (2017). The Impact of TP53 Mutations and TP53 Deletions on Survival Varies between AML, ALL, MDS and CLL: An Analysis of 3307 Cases. Leukemia.

[20] Yu Y., Xie Y., Cao L., Yang L., Yang M., Lotze M.T., Zeh H.J., Kang R., Tang D. (2015). The Ferroptosis Inducer Erastin Enhances Sensitivity of Acute Myeloid Leukemia Cells to Chemotherapeutic Agents. Mol. Cell. Oncol..

[21] Stockwell B.R., Friedmann Angeli J.P., Bayir H., Bush A.I., Conrad M., Dixon S.J., Fulda S., Gascon S., Hatzios S.K., Kagan V.E. (2017). Ferroptosis: A Regulated Cell Death Nexus Linking Metabolism, Redox Biology, and Disease. Cell.

[22] Alves L.R., Costa E.S., Sorgine M.H.F., Nascimento-Silva M.C.L., Teodosio C., Bárcena P., Castro-Faria-Neto H.C., Bozza P.T., Orfao A., Oliveira P.L. (2011). Heme-Oxygenases during Erythropoiesis in K562 and Human Bone Marrow Cells. PLoS ONE.

[23] Yang Z., Keel S.B., Wood B.L., Scott B.L., Abkowitz J.L. (2015). Pathophysiology of Macrocytic Anemia in Diamond Blackfan Anemia and Del(5q) Myelodysplastic Syndrome. Blood.

[24] Huang N.-J., Lin Y.-C., Lin C.-Y., Pishesha N., Lewis C.A., Freinkman E., Farquharson C., Millán J.L., Lodish H. (2018). Enhanced Phosphocholine Metabolism Is Essential for Terminal Erythropoiesis. Blood.

[25] Chen L.-L., Wang W.-J. (2021). P53 Regulates Lipid Metabolism in Cancer. Int. J. Biol. Macromol..

[26] Calzada E., Onguka O., Claypool S.M. (2016). Phosphatidylethanolamine Metabolism in Health and Disease. Int. Rev. Cell Mol. Biol..

[27] Allen D.W., Manning N. (1994). Abnormal Phospholipid Metabolism in Spur Cell Anemia: Decreased Fatty Acid Incorporation Into Phosphatidylethanolamine and Increased Incorporation Into Acylcarnitine in Spur Cell Anemia Erythrocytes. Blood.

[28] Luo X., Gong H.-B., Gao H.-Y., Wu Y.-P., Sun W.-Y., Li Z.-Q., Wang G., Liu B., Liang L., Kurihara H. (2021). Oxygenated Phosphatidylethanolamine Navigates Phagocytosis of Ferroptotic Cells by Interacting with TLR2. Cell Death Differ..

[29] Jiang L., Kon N., Li T., Wang S.J., Su T., Hibshoosh H., Baer R., Gu W. (2015). Ferroptosis as a P53-Mediated Activity during Tumour Suppression. Nature.

[30] Liu Y., Gu W. (2022). P53 in Ferroptosis Regulation: The New Weapon for the Old Guardian. Cell Death Differ..

[31] Xie Y., Zhu S., Song X., Sun X., Fan Y., Liu J., Zhong M., Yuan H., Zhang L., Billiar T.R. (2017). The Tumor Suppressor P53 Limits Ferroptosis by Blocking DPP4 Activity. Cell Rep..

[32] Wang J., Liu W., Zhang L., Zhang J. (2023). Targeting Mutant P53 Stabilization for Cancer Therapy. Front. Pharmacol..

[33] Wang S.J., Li D., Ou Y., Jiang L., Chen Y., Zhao Y., Gu W. (2016). Acetylation Is Crucial for P53-Mediated Ferroptosis and Tumor Suppression. Cell Rep..

[34] Matyash V., Liebisch G., Kurzchalia T.V., Shevchenko A., Schwudke D. (2008). Lipid Extraction by Methyl-*Tert*-Butyl Ether for High-Throughput Lipidomics. J. Lipid Res..

[35] Koelmel J.P., Kroeger N.M., Ulmer C.Z., Bowden J.A., Patterson R.E., Cochran J.A., Beecher C.W.W., Garrett T.J., Yost R.A. (2017). LipidMatch: An Automated Workflow for Rule-Based Lipid Identification Using Untargeted High-Resolution Tandem Mass Spectrometry Data. BMC Bioinform..

[36] Tian M. (2022). lipidmapsR: Lipid Maps Rest Service.

[37] Conroy M.J., Andrews R.M., Andrews S., Cockayne L., Dennis E.A., Fahy E., Gaud C., Griffiths W.J., Jukes G., Kolchin M. (2024). LIPID MAPS: Update to Databases and Tools for the Lipidomics Community. Nucleic Acids Res..

[38] Liebisch G., Fahy E., Aoki J., Dennis E.A., Durand T., Ejsing C.S., Fedorova M., Feussner I., Griffiths W.J., Köfeler H. (2020). Update on LIPID MAPS Classification, Nomenclature, and Shorthand Notation for MS-Derived Lipid Structures. J. Lipid Res..

[39] Fahy E., Sud M., Cotter D., Subramaniam S. (2007). LIPID MAPS Online Tools for Lipid Research. Nucleic Acids Res..

[40] Kopczynski D., Hoffmann N., Peng B., Ahrends R. (2020). Goslin: A Grammar of Succinct Lipid Nomenclature. Anal. Chem..

[41] Livak K.J., Schmittgen T.D. (2001). Analysis of Relative Gene Expression Data Using Real-Time Quantitative PCR and the 2^−ΔΔCT^ Method. Methods.

[42] Ooms J. (2014). The Jsonlite Package: A Practical and Consistent Mapping Between JSON Data and R Objects. arXiv.

[43] Villanueva R.A.M., Chen Z.J. (2019). ggplot2: Elegant Graphics for Data Analysis (2nd ed.). Meas. Interdiscip. Res. Perspect..

[44] Wickham H., François R., Henry L., Müller K., Vaughan D., Software P. (2023). PBC Dplyr: A Grammar of Data Manipulation. https://dplyr.tidyverse.org/.

[45] Graves S., Dorai-Raj H.-P.P., Selzer L., Dorai-Raj S. (2024). Multcompview: Visualizations of Paired Comparisons. https://rdrr.io/cran/multcompView/.

[46] Wickham H., Vaughan D., Girlich M., Ushey K., Software P. (2024). PBC Tidyr: Tidy Messy Data. https://tidyr.tidyverse.org/.

[47] Wickham H., Averick M., Bryan J., Chang W., McGowan L.D., François R., Grolemund G., Hayes A., Henry L., Hester J. (2019). Welcome to the Tidyverse. J. Open Source Softw..

[48] Wickham H., Henry L., Software P. (2025). PBC [cph; fnd Purrr: Functional Programming Tools. https://purrr.tidyverse.org/.

[49] The R Core Team (2018). R Core Team R: A Language and Environment for Statistical Computing.

